# Identifying Genetic Variants for Heart Rate Variability in the Acetylcholine Pathway

**DOI:** 10.1371/journal.pone.0112476

**Published:** 2014-11-10

**Authors:** Harriëtte Riese, Loretto M. Muñoz, Catharina A. Hartman, Xiuhua Ding, Shaoyong Su, Albertine J. Oldehinkel, Arie M. van Roon, Peter J. van der Most, Joop Lefrandt, Ron T. Gansevoort, Pim van der Harst, Niek Verweij, Carmilla M. M. Licht, Dorret I. Boomsma, Jouke-Jan Hottenga, Gonneke Willemsen, Brenda W. J. H. Penninx, Ilja M. Nolte, Eco J. C. de Geus, Xiaoling Wang, Harold Snieder

**Affiliations:** 1 Interdisciplinary Center Psychopathology and Emotion regulation (ICPE), Department of Psychiatry, University of Groningen, University Medical Center Groningen, Groningen, The Netherlands; 2 Department of Epidemiology, University of Groningen, University Medical Center Groningen, Groningen, The Netherlands; 3 Departments of Biostatistics & Epidemiology, College of Public Health, University of Kentucky, Lexington, Kentucky, United States of America; 4 Georgia Prevention Institute, Department of Pediatrics, Georgia Regents University, Augusta, Georgia, United States of America; 5 Department of Vascular Medicine, University of Groningen, University Medical Center Groningen, Groningen, The Netherlands; 6 Division of Nephrology, Department of Internal Medicine, University of Groningen, University Medical Center Groningen, Groningen, The Netherlands; 7 University of Groningen, University Medical Center Groningen, Department of Cardiology, Groningen, The Netherlands; 8 VU University Medical Center, Amsterdam, The Netherlands; 9 Department of Biological Psychology, EMGO institute for Health and Care research, VU University & VU Medical Center, Amsterdam, The Netherlands; Universite de Montreal, Canada

## Abstract

Heart rate variability is an important risk factor for cardiovascular disease and all-cause mortality. The acetylcholine pathway plays a key role in explaining heart rate variability in humans. We assessed whether 443 genotyped and imputed common genetic variants in eight key genes (*CHAT, SLC18A3, SLC5A7, CHRNB4, CHRNA3, CHRNA, CHRM2* and *ACHE*) of the acetylcholine pathway were associated with variation in an established measure of heart rate variability reflecting parasympathetic control of the heart rhythm, the root mean square of successive differences (RMSSD) of normal RR intervals. The association was studied in a two stage design in individuals of European descent. First, analyses were performed in a discovery sample of four cohorts (n = 3429, discovery stage). Second, findings were replicated in three independent cohorts (n = 3311, replication stage), and finally the two stages were combined in a meta-analysis (n = 6740). RMSSD data were obtained under resting conditions. After correction for multiple testing, none of the SNPs showed an association with RMSSD. In conclusion, no common genetic variants for heart rate variability were identified in the largest and most comprehensive candidate gene study on the acetylcholine pathway to date. Future gene finding efforts for RMSSD may want to focus on hypothesis free approaches such as the genome-wide association study.

## Introduction

Heart rate variability (HRV) is the variation over time of the interval between consecutive heart beats. HRV in the respiratory frequency range is a specific marker of parasympathetic control of the heart rhythm [1], and can be reliably assessed by the root mean square of successive differences (RMSSD) of normal RR intervals [2]. Prior research has produced an extensive list of HRV correlates including a broad range of somatic and mental health problems [3]. High HRV is a sign of good adaptability, often used as a marker of well-functioning cardiac autonomic control mechanisms. Reduced HRV is a predictor of hypertension [4], all-cause mortality, arrhythmic events, and sudden death after acute myocardial infarction as well as in the general population [4]–[7].

The parasympathetic (vagal) branch of the autonomic nervous system has an inhibitory influence on the pace-making activity of the sinoatrial node of the heart. Parasympathetic regulation of the heart is mediated by acetylcholine neurotransmission. Acetylcholine activates mainly two types of receptors, the muscarinic and nicotinic receptors. Muscarinic receptors are found on all effector cells that are stimulated by the postganglionic cholinergic neurons of the parasympathetic nervous system. Nicotinic receptors are found on the postganglionic neurons of the autonomic ganglia. The two types of receptors have different functions, and specific drugs can be used to stimulate or block one or the other type [8].

Although there is consistent evidence for the influence of genetic factors on HRV from twin studies showing heritabilities up to 51% [9]–[11], very few studies have tried to identify the genetic polymorphisms responsible for this heritability [12]. Genes involved in the regulatory pathways of acetylcholine, the neurotransmitter of the parasympathetic nervous system, seem plausible candidates to harbor such polymorphisms. In the present study we examined eight key genes (listed in Table 1) involved in biosynthesis, transport, breakdown, and receptor binding of acetylcholine. With the exception of the choline transporter [13], these genes have not been investigated in genetic studies of HRV before.

**Table 1:** Eight key genes involved in synthesis, metabolism and receptor binding of acetylcholine.

Protein	Gene	Chr	Physical position (bp)*	Genotyped tSNPs**	Imputed SNPs
Acetylcholinesterase	*ACHE*	7	100,487,615–100,494,594	4/4/1	3/3/6
Choline transporter	*SLC5A7*	2	108,602,979–108,630,450	12/12/16	43/43/39
Choline acetyltransferase	*CHAT*	10	50,817,141–50,901,925	26/29/19	63/60/70
Vesicular acetylcholinetransporter	*SLC18A3*	10	50,818,347–50,820,765		
Nicotinic receptors a3 subunit	*CHRNA3*	15	78,885,394–78,913,637	10/9/10	66/67/66
Nicotinic receptors a5 subunit	*CHRNA5*	15	78,857,862–78,887,611		
Nicotinic receptors β4 subunit	*CHRNB4*	15	78,916,461–79,020,096		
Muscarinic receptor 2	*CHRM2*	7	136,553,416–136,705,002	35/36***/45	181/180/171

Note: Chr, chromosome.bp, base pair;

*Physical position is based on NCBI build 35;

**GA/(TRAILS-P & TWINS)/NESDA, as TRAILS-P and TWINS were jointly genotyped the middle number represents genotyped SNPs of both cohorts. See the method section for further details;

***One tSNP was replaced with two substitutes during the assay design stage. All imputed SNPs had at least an imputation quality (Info score) of 0.5. See the methods section for further details.

Neumann et al. [13] genotyped one single nucleotide polymorphism (SNP, rs3731683) in *SLC5A7,* and observed that this polymorphism was significantly associated with HRV in 413 white adults. The *ACHE*, *CHAT,* and *SLC18A13* genes have been investigated as functional candidates for Alzheimer’s disease [14], [15]. The fact that patients with Alzheimer’s disease have reduced HRV [16], [17] and this autonomic dysfunction can be normalized by acetylcholinesterase inhibitor treatment [16] suggests that these three genes are also candidate genes for HRV. *SLC18A3* lies within the first intron of *CHAT* [18].

The genes for the β4, α3, and α5 subunits of nicotinic receptors (*CHRNB4*, *CHRNA3*, and *CHRNA5*) are clustered on chromosome 15q24 and physically linked [19]. Interestingly, the only genome-wide linkage scan conducted so far for HRV [20] yielded a multipoint LOD score of 1.84 on chromosome 15 close to this gene cluster. Among the five subtypes of muscarinic receptors (M1-M5), the mammalian heart predominantly expresses M2 receptors [21]. In *CHRM2* knockout mice, bradycardia caused by vagal stimulation was completely abolished [22].

In the current study, we assessed whether common genetic variants in these eight key genes of the acetylcholine pathway were associated with RMSSD in a total of 3429 individuals of European descent from four cohorts. Findings were replicated in 3155 subjects from three further cohorts of European descent. The key objective of this study was to identify genetic variants in the acetylcholine pathway contributing to variation in RMSSD.

## Methods

### Subjects

For the discovery stage, we included subjects from four cohorts: the Georgia cohort (GA) [23], the TRacking Adolescents’ Individual Lives Survey population cohort (TRAILS-P) [24], the Twin Interdisciplinary Neuroticism Study (TWINS) [25], and the Netherlands Study of Depression and Anxiety (NESDA) cohort [26]. As replication samples The Prevention of Renal and Vascular End-stage Disease study (PREVEND) [27], the TRacking Adolescents’ Individual Lives Survey clinical cohort (TRAILS-CC) [24], and the Netherlands Twin Registry (NTR) [28] were used. A short description of the different cohorts is given below; the general characteristics are listed in Table 2. The study protocol was approved by the Research Ethics Board at The Institutional Review Board at the Medical College of Georgia, USA (GA), Central Committee on Research Involving Human Subjects (CCMO; TRAILS-P, TRAILS-CC), Medical Ethics Committee of the University Medical Center Groningen, Groningen (TWINS, PREVEND), and the Ethical Review Board of the VU University Medical Center (NTR, NESDA). Participants were treated in accordance with the guidelines of the declaration of Helsinki, and all measurements were carried out with their adequate understanding and written consent provided by all subjects and by parents if subjects were <18 years.

**Table 2:** General characteristics of the subjects included in the study of the four discovery cohorts and the three replication cohorts.

	Discovery Cohorts				Replication Cohorts		
	GA	TRAILS-P	TWINS	NESDA	PREVEND	TRAILS-CC	NTR
N	682	1029	216	1502	2793	307	211
Men, %	50.6	47.6	0	31.4	50.5	69.3	31.2
Age (years)	19.9±4.4	11.5±0.53	23.2±3.5	41.6±12.4	53.21±11.67	11.11±0.48	30.83±14.42
RMSSD (ms)	73.8±38.6	75.5±49.9	39.0±26.3	39.9±25.8	29.35±19.70	88.77±54.26	61.02±34.41
log RMSSD	4.16±0.55	4.10±0.69	3.49±0.58	3.51±0.60	3.23±0.51	4.30±0.63	4.11±3.54

Means and SD are given unless indicated otherwise.

Note: details on the different cohorts are given in the method section.

The GA cohort included subjects from two studies: the BP Stress Study and the Georgia Cardiovascular Twin Study. Both are ongoing longitudinal studies, which have followed the subjects more than 10 years including roughly equal numbers of Caucasians and African Americans and men and women. The BP Stress Study was established in 1989 with subjects aged 7–16 years at baseline [29], while the Georgia Cardiovascular Twin Study was established in 1996 with subjects aged 7–25 years at baseline [30]. For the twin study, the twin pairs were reared together and zygosity was determined using five standard microsatellite markers. Subjects in both studies were recruited from the southeastern United States and were overtly healthy and free of any acute or chronic illness based on parental report. Study design, selection criteria and the criteria to classify subjects as Caucasians or African Americans for these two studies have been described before [31], [32]. A total of 682 Caucasians were available for this study of which i) 261 subjects (mean±SD age, 23.1±2.88 years; range, 15.6 to 31.8 years) from the BP stress cohort who had RMSSD measured on visit 13 and, ii) 421 twins (mean±SD age, 17.9±3.95 years; range, 11.9 to 32.9 years; 45 singletons and 188 twin pairs) from the Georgia Cardiovascular Twin Study who had RMSSD data available from the third visit.

The TRAILS-P cohort included subjects from the ongoing longitudinal population cohort, which started in 2001. Sample selection procedures and methods of study have been described before [24], [33], [34]. For the current study, data of 1029 10-to-13-year-old Dutch preadolescents (47.6% boys, mean±SD age, 11.5±0.53 years) were used for whom RMSSD data were available from the first measurement wave. A more detailed description of the group that participated in the cardiovascular measurements has been given elsewhere [35].

The TWINS cohort was drawn from the Groningen Twin Register, a 3-wave study including approximately 800 twin pairs from the Northern part of the Netherlands [25]. At the second wave a selection of 125 female twin pairs between 18 and 30 years old were invited to visit a psychophysiological laboratory to perform various rest and mental stress tasks. For the current study, valid RMSSD data of 216 subjects were available [11]. Zygosity of this target group was determined using 10 microsatellite markers.

The NESDA cohort was drawn from a larger 8-year longitudinal study conducted among 652 persons without depression or anxiety disorder and 2329 with a remitted or current diagnosis of depression or an anxiety disorder [26]. For the current study, 1502 NESDA subjects were included from which RMSSD data were collected on the baseline measurement session of the study and who had been genotyped on a genome-wide platform.

The PREVEND cohort was drawn from a larger community-based prospective cohort which was originally initiated to investigate the natural course of increased albuminuria levels and its association to renal and cardiovascular disease [27], [36]. PREVEND subjects completed the baseline survey from 1997 to 1998. At the second screening (2001 to 2003) RMSSD data were obtained from 2793 subjects.

The TRAILS-CC cohort included subjects from the ongoing longitudinal cohort of clinically referred subjects following the same protocol as the TRAILS-P cohort described above. Sample selection procedures and methods of study have been described before [24], [33], [37]. For the current study data of 307 TRAILS-CC 10-to-12-year-old Dutch preadolescents (69.3% boys, mean±SD age, 11.11±0.48 years) were used for whom RMSSD data were obtained at the first measurement wave.

The NTR cohort that was established in the late 1980s, consisted of Dutch adult twins (mean age ±SD, 30.83±14.42 years) that had participated in one of various studies of cardiovascular regulation [38], [39] and had been part of the GAIN genotyping effort [40]. RMSSD data obtained during a resting condition were used.

In all cohorts subjects with heart disease and those taking antidepressant or anticholinergic medications were excluded from the analyses.

### Heart rate variability measurement and processing

For the GA cohorts, the continuous RR intervals were recorded with BioZ (CardioDynamics, San Diego, CA) impedance monitors. Four dual sensors connected to the BioZ were placed on the subject’s neck and thorax, which formed four electrocardiogram (ECG) vectors. These ECG vectors can be detected by the BioZ. The BioZ then converted the RR interval into beat-to-beat heart rate with a precision of 2 decimal places, including a record of the real time of each beat. At the 10th, 12th, and 14th minute of the 15-minute resting period, a 30-second continuous period of RR intervals was recorded. Prior to calculation of HRV parameters, the raw RR interval data were firstly processed for artificial readings using the following two criteria: (1) RR intervals were between 300 and 2000 ms; (2) the successive RR interval ratios were between 0.8 and 1.2. Dedicated HRV software (Kubios HRV analysis) [41] was used to compute the RMSSD from the RR intervals. RMSSD was calculated as the average of the three 30-second segments of continuous RR intervals.

In the TRAILS-P and TRAILS-CC cohorts, RMSSD was computed on the ECG-derived RR interval time series. ECG measurements took place in a quiet room at school (TRAILS-P) or at the hospital research center (TRAILS-CC). First, subjects lied down and were encouraged to relax and not to move or speak. Next, the procedure was explained to them and data acquisition did not commence until the signal was considerably stable (i.e., as visually observed through the levelling out of the signals after initial fluctuations), usually within five minutes. RR intervals were registered for four minutes in the supine position. To obtain RR intervals with sufficient resolution for HRV determination, a special interpolation algorithm was used, increasing the time resolution for R-peak detection by a factor of 2.5. After initial visual inspection and exclusion of unusable signals, stationarity of the time series was checked and artifacts were corrected using the CARSPAN program (IECProgamma, Rijks Universiteit Groningen, Groningen, The Netherlands) [42]. A more detailed description of cardiovascular data assessment, analysis, attrition, and internal reliability has been given before [35].

In the TWINS cohort RMSSD was computed on the ECG-derived RR interval time series, which were assessed in a quiet laboratory with a three-lead ECG during four standardized conditions: a rest condition, two stress conditions, and a second rest condition. Cardiovascular measurements started after the subjects had relaxed in sitting position for at least 10 minutes. Each condition lasted for about five minutes. The average of the two rest conditions was used in the current study. The same data cleaning procedure as described above for the TRAILS-P and TRAILS-CC studies was used. Further details have been described before [43], [44].

In the NESDA cohort RMSSD was computed on the ECG-derived RR interval time series measured with the Vrije Universiteit Ambulatory Monitoring System (VU-AMS) [45], [46]. Subjects wore the VU-AMS device during a large part of the baseline assessment while participating in other assessments (medical examination, an interview and a computer task). Further details on the assessment have been described before [47].

In the PREVEND cohort RMSSD was computed on the RR interval time series based on the beat-to-beat blood pressure signals measured with the Portapres device (FMS Finapres Medical systems BV, Amsterdam, The Netherlands; [48]). Subjects were measured in the supine resting condition, after resting for five minutes, during 15 minutes, while holding their hand with the Portapres cuff at heart level. They were not allowed to talk or to move during the measurement. In short, from the 15 minute recording an artifact free period of at least 100 seconds but no longer than five minutes was selected for RMSSD calculations. The same data cleaning procedure as described above for the TRAILS-P, TRAILS-CC, and TWINS studies was used. Further details have been described before [49].

In the NTR cohort RMSSD was computed on the ECG-derived RR interval time series measured with the VU-AMS device during a 24-h ambulatory monitoring procedure which has been described before [38], [39]. Prompted by the VU-AMS device, the subjects filled out a diary every 30 (±10) minutes in which they logged their physical activities and bodily postures since the previous prompt. This diary information was combined with simultaneously recorded vertical accelerometer data to divide the entire 24-h recording into periods of homogeneous activities/postures (median duration 26.2 minutes). From the total set of ambulatory RMSSD measurements only the evening measurements were selected where subjects were seated calmly for at least five minutes but no longer than 60 minutes. The average of these measurements was taken as their RMSSD resting value.

### SNP selection

For the *GA* cohorts, tagging SNPs were selected from HapMap phase II data (July, 2007) for seven genes including *CHAT, SLC18A3, SLC5A7, CHRNB4, CHRNA3, CHRNA5* and *CHRM2* using Haploview pairwise tagging with a r^2^≥0.8. All the common SNPs with a minor allele frequency (MAF) ≥5% within the gene as well as −10 kb upstream and +10 kb downstream of the gene were covered. Since the HapMap had a limited number of SNPs available in the *ACHE* gene at the time when tagging SNPs were selected (July, 2007), the SNP data from the SeattleSNPs Variation Discovery Resource (www.pga.gs.washington.edu), which had completely re-sequenced *ACHE* in 23 white Americans, was used for tagging SNPs selection. Table 1 lists the physical position, number of the tagging SNPs selected, the genotyped and imputed SNPs for the eight key genes. Since *SLC18A3* is within the first intron of the *CHAT* gene and *CHRNB4*, *CHRNA3,* and *CHRNA5* are within one gene cluster, these genes were listed together as two loci in Table 1.

For the TRAILS-P, TRAILS-CC and TWINS cohorts, the same tagging SNPs as selected for the Caucasian GA cohorts described above were selected. For the NESDA cohort, which had genome wide genotyping data, SNPs within the same physical regions were selected for this study.

### DNA extraction and genotyping

In the GA cohorts, genotyping of blood derived DNA was performed by BioServe Biotechnologies (Laurel, MD) using Sequenom’s high-throughput matrix-assisted laser desorption/ionization time-of-flight (MALDI-TOF) mass spectrometry. All genotyping results were reviewed manually for quality control. Two ‘no template’ controls and two positive controls were included on each plate. In addition, 107 sets of blinded controls were distributed throughout the plates for quality control purposes. There was excellent observer agreement in the randomly selected duplicates that were included for quality control purposes. The kappa statistic ranged from 0.89 to 1, with an average of 0.974.

In a subsample of the TRAILS-P, TRAILS-CC and TWINS cohorts, DNA was extracted from blood samples or (in a few cases) buccal swabs (Cytobrush) using a manual salting out procedure [50]. Genotyping was performed on the Golden Gate Illumina BeadStation 500 platform (Illumina Inc., San Diego, CA, USA), following the manufacturers protocol. Genotyping data and clustering was performed in BeadStudio 3.0 (Illumina Inc., San Diego, CA, USA). Concordance between DNA replicates showed a genotyping accuracy of 100%. Data cleaning was in line with standard procedures [51]. The call rate in the combined set of TRAILS-P, TRAILS-CC, and TWINS after data cleaning was 99.8%. In addition to this candidate gene genotyping, genome-wide genotyping of TRAILS-CC was performed using the Illumina HumanCytoSNP12 v2 beadchip assay (Illumina, Inc; San Diego, CA, USA). Genotypes were called with the Illumina GenomeStudio software package (Illumina, Inc). Details on genotyping and quality control have been given elsewhere [52].

In the NESDA and NTR cohorts, genome-wide genotyping of a subsample of the subjects was performed as part of the GAIN initiative to detect genetic variation associated with major depression [40]. Genotyping was performed on Affymetrix 500 K GeneChip arrays. A detailed description of genotyping and quality control criteria has been given previously [53].

For PREVEND genome-wide genotyping of a subsample of the cohort was performed using the Illumina HumanCytoSNP12 v2 beadchip assay (Illumina, Inc; San Diego, CA, USA). Genotypes were called with the Illumina GenomeStudio software package (Illumina, Inc). Details on genotyping and quality control have been described before [54].

### Imputation

Non-genotyped SNPs within the eight genes, as well as −10 kb upstream and +10 kb downstream of the genes, were imputed. For the GA, TRAILS-P and TWINS cohorts, for seven genes (*CHAT, SLC18A3, SLC5A7, CHRNB4, CHRNA3, CHRNA5* and *CHRM2*), imputation was performed using Phase II CEU HapMap data (release 24, build 36) as the reference database using IMPUTE version 0.3.2[55], while for the *ACHE* gene, imputation was performed using Phase III CEU HapMap data (release 2, build 36) because coverage of this gene in HapMap Phase II (release 24) was minimal. For the NESDA cohort imputation was done with IMPUTE version 2.1.2 [56] using the ALL panel of the 1000 Genomes phase I integrated variant set of March 2012. For the NTR and TRAILS-CC cohorts imputation was done with IMPUTE version 0.3.2 [55] using Phase II CEU HapMap data (release 22, build 36) reference population. For the PREVEND cohort imputation was done with Beagle version 3.1.0 [57] using the Phase II CEU HapMap (release 23a, build 36) reference population.

### Statistical analysis

Hardy-Weinberg equilibrium (HWE) was tested in each cohort by a χ^2^ test with 1 df. To prevent inflated significance, these tests were performed in data including only one of the twins, chosen at random, in the GA and TWINS cohorts. For the GA cohort, SNPs were excluded if they violated HWE (p<0.01) and at the same time the kappa statistic for the randomly selected duplicates on the genotype of these SNPs was less than 0.90. For the TRAILS-P and TWINS cohorts, SNPs were excluded if they violated HWE (p<0.01). For the NESDA, NTR, PREVEND, and TRAILS-CC cohorts SNPs with a HWE p-value below10^−5^ were excluded.

The genotyped and imputed SNPs were examined for their associations with RMSSD under an additive model of inheritance with adjustment for age and sex. For imputed SNPs dosages were calculated and used in the analyses to properly account for uncertainty in the imputed genotypes. RMSSD was log-transformed in the analyses. For the GA and TWINS cohorts, generalized estimating equations were used with exchangeable correlation structure to account for familial correlations. A fixed effects meta-analysis on these four cohorts was conducted using the inverse variance weighted method. The Q and I^2^ statistics were used to estimate between study heterogeneity. To help interpret I^2^’s magnitude we used guidelines from Higgins and colleagues [58] in which I^2^≤25% marks low, I^2^ = 50% medium, and I^2^≥75% high heterogeneity.

To optimize the chances of discovery we used a relatively lenient p<0.05 cut-off in the discovery stage. That is, all SNPs with p<0.05 in the discovery stage were taken forward for replication in three independent samples: PREVEND, TRAILS-CC and NTR. For all three replication cohorts results for these SNPs were extracted from genome-wide analyses of log-transformed RMSSD, who analyzed it by means of SNPTEST [55] using an additive model with age and sex as covariates and taking the uncertainty in the imputed genotypes into account. Results from discovery and replication phase were subsequently combined in a meta-analysis using METAL [59]. When not mentioned analyses were performed in StataSE software (version 12, StataCorp, College Station, Texas, USA).

## Results

The characteristics of the subjects included in the analysis in each of the cohorts are given in Table 2. Of the 95 SNPs selected in the design stage, 87 were successfully genotyped in the GA cohort and 90 in the TRAILS-P and TWINS cohorts (Table 1). No further SNPs were excluded as all met our criteria for inclusion (see methods). All imputed SNPs in the discovery cohorts had at least an imputation quality (info score) of 0.5. In total, 443 genotyped and imputed SNPs were included in the association analysis with RMSSD.

### Discovery stage

In the discovery phase the association between the candidate SNPs and RMSSD were tested in four cohorts (n = 3429; GA, TRAILS-P, TWINS and NESDA). For 25 SNPs an association with RMSSD (p<0.05) was found (see Table 3).

**Table 3:** Results of discovery stage, replication stage and the overall meta-analysis of the 25 SNPs associated with RMSSD (p<0.05) in the discovery stage.

	Gene	SNP	C/NC	Discovery						Replication						Overall meta-analysis				
				CAF	Beta	SE	Pvalue	I^2^ (%)	PvalueQstatistic	CAF	Beta	SE	P value	I^2^ (%)	Pvalue Q statistic	Beta	SE	Pvalue	I^2^ (%)	Pvalue Qstatistic
1	SLC5A7	rs3731683	G/A	0.66	−0.052	0.016	0.002	14.9	0.32	0.67	0.048	0.024	0.044	0	0.48	−0.020	0.013	0.144	91.6	0.001
2	SLC5A7	rs333214	A/G	0.88	−0.061	0.022	0.005	18.4	0.30	0.87	0.031	0.057	0.583	37.9	0.20	−0.049	0.020	0.016	56.6	0.13
3	CHRM2	rs17506824	T/C	0.47	−0.035	0.014	0.014	0	0.97	0.47	−0.008	0.012	0.511	0	0.48	−0.020	0.010	0.034	50.1	0.16
4	CHRM2	rs6967953	T/C	0.43	−0.035	0.015	0.017	0	0.76	0.41	−0.007	0.014	0.591	0	0.40	−0.020	0.010	0.044	47.7	0.17
5	SLC5A7	rs17269293	C/G	0.82	−0.043	0.018	0.021	75.9	0.01	0.79	0.018	0.023	0.436	0	0.83	−0.019	0.015	0.185	76	0.04
6	CHRM2	rs324654	T/C	0.07	−0.066	0.029	0.022	0	0.66	0.08	0.018	0.023	0.426	0	0.94	−0.015	0.018	0.417	80.9	0.02
7	CHRM2	rs6962027	T/A	0.49	−0.032	0.014	0.024	0	0.89	0.47	−0.009	0.013	0.473	0	0.38	−0.019	0.010	0.042	31.9	0.23
8	CHRM2	rs10246819	T/C	0.49	−0.031	0.014	0.029	0	0.92	0.47	−0.009	0.012	0.461	0	0.42	−0.019	0.010	0.047	27.7	0.24
9	CHRM2	rs324576	T/C	0.08	−0.058	0.027	0.032	0	0.62	0.08	0.012	0.021	0.600	0	0.83	−0.016	0.017	0.352	74.9	0.05
10	CHRM2	rs324578	A/T	0.08	−0.058	0.027	0.033	0	0.62	0.07	0.098	0.056	0.077	0	0.47	−0.028	0.025	0.255	84.3	0.01
11	CHRM2	rs324579	G/A	0.08	−0.058	0.027	0.034	0	0.62	0.08	0.012	0.022	0.582	0	0.76	−0.015	0.017	0.367	75.1	0.05
12	CHRM2	rs324580	T/C	0.08	−0.058	0.027	0.034	0	0.61	0.08	0.012	0.022	0.582	0	0.76	−0.015	0.017	0.368	75	0.05
13	CHRM2	rs324587	T/A	0.08	−0.057	0.027	0.034	0	0.62	0.08	0.012	0.022	0.591	0	0.70	−0.016	0.017	0.350	74.4	0.05
14	CHAT	rs10508915	A/G	0.85	−0.044	0.021	0.035	0	0.92	0.85	−0.003	0.017	0.848	0	0.52	−0.020	0.013	0.137	56.2	0.13
15	CHRM2	rs324582	G/A	0.08	−0.056	0.027	0.038	0	0.61	0.08	0.012	0.022	0.582	0	0.75	−0.015	0.017	0.382	74	0.05
16	CHRM2	rs324584	G/A	0.08	−0.056	0.027	0.039	0	0.65	0.08	0.012	0.022	0.581	0	0.75	−0.015	0.017	0.389	73.8	0.05
17	CHRM2	rs324632	C/T	0.08	−0.056	0.027	0.041	0	0.57	0.06	0.040	0.044	0.357	0	0.97	−0.029	0.023	0.214	71.3	0.06
18	SLC5A7	rs333227	G/A	0.15	−0.041	0.020	0.041	0	0.56	0.16	−0.003	0.017	0.858	49.8	0.14	−0.019	0.013	0.148	53	0.15
19	SLC5A7	rs333230	G/T	0.15	−0.041	0.020	0.042	0	0.56	0.15	0.001	0.019	0.981	50.9	0.13	−0.019	0.014	0.172	56.2	0.13
20	CHRM2	rs324612	T/C	0.08	−0.055	0.027	0.043	0	0.65	0.08	0.027	0.026	0.313	0	0.86	−0.130	0.019	0.490	78.5	0.03
21	SLC5A7	rs333226	G/A	0.15	−0.041	0.020	0.044	0	0.48	0.16	−0.005	0.017	0.768	11.7	0.32	−0.020	0.013	0.131	45.9	0.17
22	CHRM2	rs324624	A/G	0.08	−0.054	0.027	0.046	0	0.69	0.06	0.049	0.043	0.253	0	0.97	−0.025	0.023	0.280	75.7	0.04
23	CHRM2	rs324627	G/A	0.08	−0.054	0.027	0.047	0	0.69	0.07	0.021	0.026	0.424	0	0.83	−0.015	0.019	0.426	74.8	0.05
24	CHAT	rs12356649	A/G	0.85	−0.041	0.021	0.048	0	0.91	0.85	−0.003	0.017	0.852	0	0.57	−0.019	0.013	0.159	49	0.16
25	CHAT	rs12358003	G/C	0.85	−0.041	0.021	0.048	0	0.91	0.84	0.005	0.031	0.886	0	0.59	−0.027	0.017	0.117	32.4	0.22

SNPs are sorted according to p-value in the discovery cohort.

Note: RMSSD was log-transformed prior data analysis. Analyses are age & sex adjusted. Discovery stage sample size (N) = 3429; Replication stage N = 3311(except for one SNP [rs333214] in the *SLC5A7* gene where N = 516, because this SNP could not be analyzed in PREVEND due to low imputation quality); and overall meta-analysis N = 6740. See the methods section for further details on the imputation procedures for the different cohorts. C/NC, coded/non-coded allele; CAF, coded allele frequency.

When testing the genetic heterogeneity of the SNPs in this phase, the point estimate for I^2^ was significant for only one SNP (rs17269293; I^2^ = 75.8%). Heterogeneity for all other SNPs was either completely absent or low and non-significant, confirming that performing a fixed effects meta-analysis on these data is justified (Table 3).

### Replication stage and the overall meta-analysis

To validate the association found in the discovery phase, the 25 lead SNPs (Table 3), were taken forward to the replication phase in three independent cohorts (n = 3311 PREVEND, TRAILS-CC and NTR). Imputation quality was very high for NTR (all 25 SNPs: info score >0.72; 21 SNPs: info score >0.90) and TRAILS-CC (all 25 SNPs: info score >0.78; 24 SNPs: info score >0.94) and somewhat lower for PREVEND, which had 18 SNPs of high quality imputation (r^2^>0.73), but 7 SNPs with lower imputation quality (r^2^<0.35). For one of these SNPs (rs333214 in the *SLC5A7* gene) data had to be excluded from the PREVEND cohort as the imputation quality was too low (r^2^<0.10) resulting in lower replication sample size (n = 516). None of the SNPs were replicated with a nominal p<0.05, except for one SNP. However, this SNP (rs3731683) showed an effect in the opposite direction as observed in the discovery stage (see the opposite beta coefficients for this SNP in Table 3). Thus, none of the SNPs were replicated.

The overall meta-analysis combining discovery and replication stage (n = 6740) gave no significant Bonferroni corrected (p<0.0005, conservatively assuming 100 tests for the almost 100 independent tagging SNPs from Table 1) findings for any of the 25 SNPs (Table 3).

## Discussion

The current study comprehensively tested the association between all common (MAF≥5%) variants in eight key genes of the acetylcholine pathway and an established measure of HRV, the RMSSD, in subjects of European descent. The eight key genes were carefully selected in this two-stage candidate gene study on the basis of their involvement in biosynthesis, transport, breakdown, and receptor binding of acetylcholine. However, none of the common genetic variants in these eight genes of the acetylcholine pathway were significantly associated with RMSSD.

Neumann and colleagues [13] genotyped a single SNP (rs333229) in the choline transporter gene *SLC5A7* in their study of 413 individuals of European ancestry (aged 30 to 54 years). They found an association with the power in the high frequency (HF) band (HF power of Fourier transformed inter-beat-interval time series of RR intervals of the ECG, is another measure of HRV comparable to RMSSD) obtained during five minutes rest: compared with GG homozygotes, T allele carriers had *higher* HF power. In a more recent study on air pollution in 61 glucose intolerant subjects, T allele carriers of this SNP (rs333229, which was one of the 139 tested SNPs) had *lower* SDNN in response to air pollution [60]. Interestingly, the exact same SNP in *SLC5A7* (rs333229) was genotyped in the current study but did not survive our discovery stage (i.e., was not significantly associated with RMSSD). As such, the significant results in the above two underpowered studies should probably be interpreted as chance findings, because our discovery stage alone (n = 3429) already had more than eight times the sample size of the largest of those two previous studies (n = 413).

Our study has a number of strengths compared to previous candidate gene studies that investigated only a single SNP in a single gene and/or had (very) small sample sizes. We comprehensively tested 443 genotyped and imputed common SNPs in eight key genes of the acetylcholine pathway in which our selected tagging SNPs covered all SNPs with MAF≥5% and r^2^≥0.8 ranging from −10 kb upstream to +10 kb downstream of those genes. Another strong point of our study is that we aimed to replicate the initially discovered associations in independent samples. As replication failed, our initial findings should be labeled as false-positive findings, reinforcing the idea that independent replication is essential in candidate gene studies. Our two stage association study with large combined sample size of n = 6740 had more than 80% power with a Bonferroni corrected alpha of 0.0005 for 100 tests to detect SNPs explaining as little as 0.32% of the variance in RMSSD. As such it is highly unlikely that common SNPs in the investigated genes explain a considerable part of individual differences in RMSSD in rest.

A potential limitation of our study is that the RMSSD measures in the different cohorts were assessed in both sitting and supine positions, which might have introduced heterogeneity in our RMSSD data. Using these heterogeneous phenotypes might have decreased our power to detect associations somewhat. Not all SNPs were genotyped in our study, which may have limited our power to find or confirm significant associations. However, common SNPs in the discovery cohorts were well imputed (info score >0.5), because we selected tagging SNPs with good coverage of the genes and used 1000 Genomes imputation in NESDA. For the replication cohorts the imputation quality was dependent on the coverage of the GWAS chip and the imputation software used. Imputation quality was very high for NTR and TRAILS-CC, but somewhat lower for 7 SNPs in PREVEND reducing the effective sample size and power for these SNPs. We guarded against potential biases caused by the imputation process such as inflated significance, through properly accounting for uncertainty in these imputed genotypes in our analyses. Furthermore, the findings and conclusions of the current study are not generalizable to individuals of non-European descent.

Another, perhaps more compelling, reason for our null-finding is that our approach was based on currently existing knowledge about the acetylcholine pathway. Using a hypothesis-driven design is a strength, but has also clear downsides. First of all, not all known elements of the acetylcholinergic signaling cascade were considered. Second, current knowledge about the acetylcholine pathways may be vastly incomplete. Third, RMSSD is known to be a complex phenotype influenced by multiple genes [61], physiological and psychological systems, and their interactions, many of which may be outside of the acetylcholine pathways.

In conclusion, no common genetic variants for HRV were identified in the largest and most comprehensive candidate gene study on the acetylcholine pathway to date. Future gene finding efforts for RMSSD may want to focus on hypothesis free approaches such as the genome-wide association study.
